# The Role of Dermoscopy in Diagnosis: A Case Presentation of Pseudoxanthoma Elasticum Initially Misdiagnosed as Pigmented Contact Dermatitis

**DOI:** 10.5826/dpc.1103a64

**Published:** 2021-07-08

**Authors:** Ömer Faruk Elmas, Abdullah Demirbaş, Raşit Kılıç, Asuman Kilitçi

**Affiliations:** 1 Department of Dermatology, Kırıkkale University, Kırıkkale, Turkey; 2 Department of Dermatology, Evliya Çelebi Training and Research Hospital, Kütahya Health Sciences University, Kütahya, Turkey; 3 Department of Ophthalmology, Tokat Gaziosmanpaşa Unıversity, Tokat, Turkey; 4 Department of Pathology, Kırşehir Ahi Evran University, Kırşehir, Turkey

**Keywords:** pseudoxanthoma elasticum, pigmented contact dermatitis, dermoscopy

## Case Presentation

**Figure 1:**
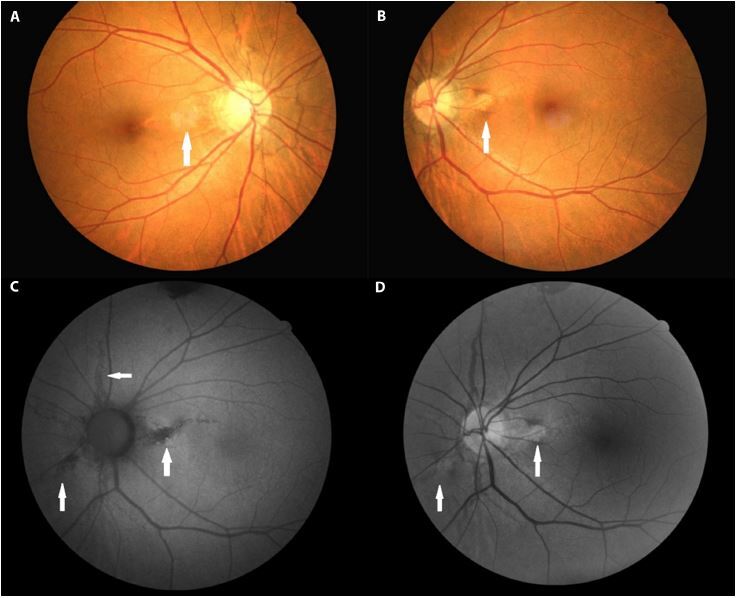
Color fundus photographs. (A, B) Angioid streaks in both eyes (white arrows). (C, D) Angioid streaks in fundus autofluorescence and red free images of the left eye (white arrows).

**Figure 2:**
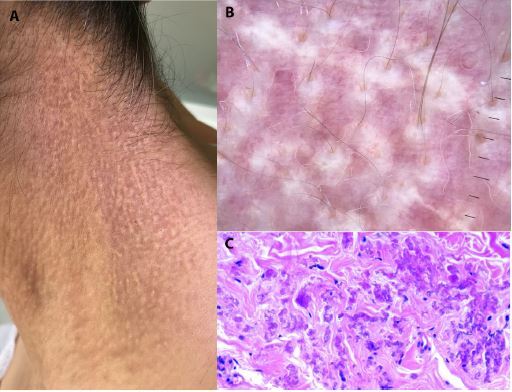
Dermatologic, dermoscopic, and histopathologic examination. (A) Dermatological examination showed confluent yellowish papules on the neck. (B) Dermoscopic examination revealed reticulated yellow-to-white clods on a light red background, along with disfocused linear irregular vessels. (C) Histopathological examination showing dermal accumulation of swollen clumped fibers.

A 45-year-old female with a three-year history of bilateral ocular angioid streaks was referred to our dermatology department Kırşehir Ahi Evran University for the evaluation of persistent cutaneous lesions, present since childhood (Figure 1, A–D). The patient was previously diagnosed with pigmented contact dermatitis 5 years ago, in a different dermatology outpatient clinic. Dermatological examination in our dermatology department showed confluent yellowish papules on both sides of the neck (Figure 2A). Dermoscopic examination revealed reticulated yellow-to-white clods on a light red background, along with defocused linear irregular vessels (Figure 2B). Histopathological examination showed mild epidermal atrophy, accumulation of swollen clumped fibers, mild inflammatory cell infiltration, and dilated dermal vessels (Figure 2C). Elastic van Gieson staining showed fragmented elastic fibers. Based on the clinical, dermoscopic and histopathological features, a diagnosis of pseudoxanthoma elasticum was made. Detailed imaging studies showed no systemic involvement except for ocular lesions.

## Teaching Point

Pseudoxanthoma elasticum has a peculiar dermoscopic pattern composed of coalescing and reticulated yellow clods on a light red background [1,2]. A thorough dermoscopic examination may prevent misdiagnosis especially in those cases characterized by subtle cutaneous manifestations.
